# Cocoa, Blood Pressure, and Vascular Function

**DOI:** 10.3389/fnut.2017.00036

**Published:** 2017-08-02

**Authors:** Valeria Ludovici, Jens Barthelmes, Matthias P. Nägele, Frank Enseleit, Claudio Ferri, Andreas J. Flammer, Frank Ruschitzka, Isabella Sudano

**Affiliations:** ^1^Cardiology, University Heart Center, University Hospital and University of Zurich, Zurich, Switzerland; ^2^Department of Life, Health and Environmental Sciences, University of L’Aquila, L’Aquila, Italy

**Keywords:** cocoa, endothelial function, blood pressure, arterial stiffness, flavonoids

## Abstract

Cardiovascular disease (CVD) represents the most common cause of death worldwide. The consumption of natural polyphenol-rich foods, and cocoa in particular, has been related to a reduced risk of CVD, including coronary heart disease and stroke. Intervention studies strongly suggest that cocoa exerts a beneficial impact on cardiovascular health, through the reduction of blood pressure (BP), improvement of vascular function, modulation of lipid and glucose metabolism, and reduction of platelet aggregation. These potentially beneficial effects have been shown in healthy subjects as well as in patients with risk factors (arterial hypertension, diabetes, and smoking) or established CVD (coronary heart disease or heart failure). Several potential mechanisms are supposed to be responsible for the positive effect of cocoa; among them activation of nitric oxide (NO) synthase, increased bioavailability of NO as well as antioxidant, and anti-inflammatory properties. It is the aim of this review to summarize the findings of cocoa and chocolate on BP and vascular function.

## Introduction

Cardiovascular disease (CVD) represents the most common cause of death in the Western world, with an estimated 17.5 million people dying from CHD (coronary heart disease or stroke) every year (1). A nutraceutical approach has been proposed to counteract the increasing burden of CVD. The consumption of polyphenol-rich foods has been related to a lower risk of cardiovascular events (cardiovascular mortality, myocardial infarction, and stroke) both in the general population and in patients with cardiovascular risk factors in several interventional and epidemiological trials (2–5). Polyphenols are believed to be largely responsible for this protective role. Characterized as compounds with phenolic structural features (6), they are a class of natural bioactive substances with numerous anti-atherogenic properties including anti-inflammatory, anti-aggregating, and vasodilatory effects, the ability to lower blood pressure (BP), to prevent oxidation of low-density lipoprotein (LDL), and to improve glucose and lipid profiles (7).

Fruits, vegetables, tea, chocolate, and wine contain a high amount of polyphenols. Among them, cocoa beans are one of the richest known sources of flavonoids (8), and their protective properties have been recognized and used by several cultures among centuries. The origins of chocolate are usually traced back to the pre-Columbian populations, which were probably the first to cultivate cocoa plants. The consumption of cocoa, appreciated for its invigorating and healthy effects, differed from today: they used to dissolve dried cocoa beans in water, adding cinnamon and pepper to enhance their strong and bitter taste. With its arrival in Europe in the sixteenth century, cocoa was processed in a sweet soft beverage and rapidly became a luxury item. In the last century, the words cocoa and chocolate became intensely linked to hypertension, diabetes, overweight, and obesity (9). However, in the last two decades the salutary cardiovascular effects of this ancient medicinal food have begun to be reevaluated, and these properties have been related to cocoa’s high content of flavonoids, members of the broader polyphenol class. The main constituents are flavanols, present as monomeric (−)-epicatechin and (+)-catechin, together with their dimers, oligomers, and polymers, the so-called proanthocyanidins, responsible for cocoa bitterness when complexing with salivary proteins (10). Although flavanols are likely to be responsible for cocoa’s beneficial effects, they are lost during the conventional chocolate manufacturing process, so that the total flavanol content of commercial chocolate varies by more than 10-folds (11). Upon harvest, cocoa beans are usually fermented by environmental microbiota. This process creates flavor precursors that further develop during the roasting step, generating the peculiar cocoa flavors that define its quality (12). Fermentation and roasting significantly decreases the polyphenol and flavanol content of cocoa due to high temperature conditions and oxidation (13). Furthermore, alkalinization, used to modify cocoa color and give it a milder taste, results in a 60% decrease of total flavanol content (14). In humans, flavanol serum concentration increases in a dose-dependent manner after ingestion, reaching its peak usually 2–3 h after cocoa intake (15, 16). Flavanols are still detectable in plasma 8 h after consumption (17). Cocoa is also rich in theobromine, a 3,7-dimethylated xanthine alkaloid, and minerals such as potassium or magnesium (18).

Several epidemiological (19–21) and interventional studies strongly suggest that cocoa consumption, as well as vegetables and fruit intake, has numerous beneficial effects on cardiovascular health, including lowering of BP (22), improving vascular function (23), reducing of platelet aggregation and adhesion (24), having anti-inflammatory properties (25), and improving glucose and lipid metabolism (26). These positive effects have been found in healthy subjects (27) as well as in patients with risk factors (arterial hypertension, diabetes, and smoking) (28) or established CVD (coronary heart disease or heart failure) (29). Various potential mechanisms, including the increased bioavailability of nitric oxide (NO) and the anti-inflammatory and antioxidant effect, are supposed to be responsible for the protective properties of cocoa (30).

This review aims to summarize the effects of cocoa and chocolate on BP and vascular function.

## Epidemiological Evidence

After observing the Kuna Indians from Central America, researchers discovered how cocoa might be able to lower BP and extend life expectancy (31). Indeed, this population surprisingly had a very low incidence of hypertension when aging, despite having a high salt diet compared to other normotensive communities (32). Interestingly, this was not related to genetic factors, since the same population, migrated from the San Blas islands to Panama City for economic reasons, showed to have BP levels similar to other urban dwelling people (31). In addition, when compared to other American citizens, a marked reduction in cardiovascular mortality was noticed (33). To explain this difference, many environmental factors were investigated, such as differences in lifestyle or tobacco use, but ruled out as contributories. Finally, it was found that Island-Kuna, but not Mainland-Kuna, used to drink five cups of cocoa per day, which, moreover, was determined to be flavonoid-rich (approximate intake 900 mg per day, i.e., the suggested highest intake worldwide) (34).

Since then, many epidemiological studies confirmed the assumption that cocoa could be responsible for these findings. Flavonoid-rich foods intake, and particularly chocolate consumption, were associated with a lower risk of death due to CVD in the Iowa Women’s Health Study (19). Subsequently, in the Zutphen Elderly Study, a similar reduction in cardiovascular mortality after chocolate consumption was reported. Comparing the groups with higher and lower chocolate intake, a reduction of 3.7 mmHg in systolic BP (SBP; 95% CI, −7.1 to −0.3 mmHg; *p* = 0.03) and a reduction of 2.1 mmHg in diastolic BP (DBP; 95% CI, −4.0 to −0.2 mmHg; *p* = 0.03) were observed. Higher chocolate intake was associated to significant reduction in cardiovascular mortality (adjusted relative risk 0.50, 95% CI, 0.32–0.78; *p* = 0.004) and all-cause mortality (adjusted relative risk 0.53, 95% CI, 0.39–0.72; *p* < 0.001) (20). In the Stockholm Heart Epidemiology Program, chocolate intake related to a reduced cardiovascular mortality after acute myocardial infarction (21). Furthermore, this reduction appeared to be dose dependent. Compared with those never consuming chocolate, the subjects consuming chocolate less than once, up to once or up to twice per week showed progressively decreasing hazard ratios for cardiac mortality [0.73 (95% CI, 0.41–1.31), 0.56 (0.32–0.99), and 0.34 (0.17–0.70), respectively]. However, chocolate consumption was weakly associated with a lower rate of total mortality and non-fatal outcomes (21). The same findings emerged in two cohort studies of middle-aged Swedish women and men, in which daily moderate chocolate intake had an inverse association with chronic heart failure hospitalization and death (35, 36). Furthermore, in the National Heart, Lung, and Blood Institute Family Heart Study, chocolate intake correlated inversely with prevalent coronary heart disease in a general United States population (37). The most recent epidemiological data coming from the analysis of the European Prospective Investigation into Cancer Norfolk cohort support the prior findings (38). When compared to those who consumed no chocolate, subjects in the highest quintile of chocolate intake (15.6–98.8 g per day) demonstrated a significantly reduced rate of stroke (HR 0.77, 95% CI, 0.62–0.96) and cardiovascular mortality (HR 0.75, 95% CI, 0.62–0.92). Similar results emerged from a meta-analysis of 9 separate studies involving 157,809 participants (38). However, it is important to underline that none of these epidemiological studies focused on the amount of cocoa intake. Thus, it is not possible to make efficient comparison between the abovementioned studies.

## Cocoa and BP: Interventional Studies

Arterial hypertension is a major modifiable risk factor for cardiovascular and cerebrovascular disease (39). Every 10 mmHg reduction in SBP significantly reduces the risk of major cardiovascular events, CHD, stroke, and heart failure, which leads to a significant 13% reduction in all-cause mortality (40). To date, several interventional studies have assessed the efficacy of cocoa in lowering BP both in healthy subjects and in patients with cardiovascular risk factors (Table 1).

**Table 1:** Studies investigating cocoa and blood pressure.

Reference	Year	Study design	Population	Duration (weeks)	Intervention	Reduction of SBP/DBP active group (mmHg)	Reduction of SBP/DBP control group (mmHg)
Fraga et al. (41)	2005	Randomized crossover	28 normotensive	2	High-flavanol milk (168 mg)/white chocolate	−6/−5	−2/−1
Grassi et al. (42)	2005	Randomized crossover	15 normotensive	1	Dark (500 mg flavanols)/white chocolate	−7/−4.2	−0.5/−0.3
Crews et al. (43)	2008	Randomized double-blind parallel	101 normotensive	6	Dark chocolate (397 mg flavanol) and cocoa drink (357 mg flavanol)/low-flavanol chocolate and drink	−3.58/−0.5	−3.05/−0.57
Mastroiacovo et al. (44)	2015	Randomized double-blind parallel	90 normotensive	8	High (993 mg)/intermediate (520 mg)/low (48 mg) flavanol cocoa drink	−7.83/−4.77	−1.60/−1.57
Sansone et al. (27)	2015	Randomized double-blind parallel	100 normotensive	4	High (450 mg)/low-flavanol cocoa drink	−4.4/−3.9	−1/0
Grassi et al. (23)	2015	Randomized double-blind crossover	20 normotensive	1	High (800, 500, 200, 80 mg)/low (0 mg) flavanol cocoa	−4.8/−3.03	–/–
Murphy et al. (45)	2003	Randomized double-blind parallel	32 normotensive	4	High (234 mg flavanols and procyanidins)/low-flavanol chocolate	+2/−1	+3/0
Engler et al. (46)	2004	Randomized double-blind	21 normotensive	2	High (213 mg procyanidins, 46 mg epicatechin)/low-flavanol chocolate	−1/+0.9	−2.8/−0.1
Shiina et al. (47)	2009	Randomized single-blind	39 normotensive	2	Dark (550 mg flavanols)/white chocolate	+4.6/+6.6	+4/+5.2
Njike et al. (48)	2011	Randomized crossover	44 normotensive overweight	6	High/low-flavanol cocoa drink	+2.2/−0.5	−0.1/+0.8
Taubert et al. (49)	2003	Randomized crossover	13 hypertensive	2	Dark (500 mg flavanols)/white chocolate	−5.1/−1.8	+0.4/+0.3
Taubert et al. (50)	2007	Randomized single-blind parallel	44 pre-hypertensive/hypertensive	18	Dark(30 mg flavanols)/white chocolate	−2.9/−1.9	+0.1/0
Grassi et al. (51)	2005	Randomized crossover	20 hypertensive	2	Dark (500 mg flavanols)/white chocolate	−11.9/−8.5	−0.7/−0.6
Muniyappa et al. (52)	2008	Randomized double-blind crossover	20 hypertensive	2	High (900 mg)/low-flavanol drink	−2/−3	−1/−4
Davison et al. (53)	2010	Randomized double-blind crossover	52 hypertensive	6	High (1,052 mg)/low-flavanol cocoa drink	−5.3/−3	−2.1/+0.1
Grassi et al. (54)	2008	Randomized crossover	19 hypertensive with IGT	2	Dark (1,008 mg polyphenols)/white chocolate	−3.8/−3.9	−0.1/−0.2
Davison et al. (55)	2008	Randomized parallel	49 normotensive obese or overweight	12	High (902 mg)/low-flavanol cocoa	−1.9/−1.8	+4.2/+2.8
Rostami et al. (28)	2015	Randomized double-blind	60 hypertensive diabetic	8	Dark/white chocolate	−5.9/−6.4	−1.1/+0.2
Monagas et al. (56)	2009	Randomized crossover	47 diabetics or more than 3 CV risk factors	4	Cocoa powder (495 mg polyphenols) with milk/milk	0.0/−2	−3/−3
De Palma et al. (57)	2016	Randomized crossover	32 patients with stable HF	4	High (1,064 mg)/low-flavanol dark chocolate	−1.8/−4.2	−0.9/+2.9

*IGT, impaired glucose tolerance; HF, heart failure; CHD, coronary heart disease; CV, cardiovascular; SBP, systolic blood pressure; DBP, diastolic blood pressure*.

Compared to cocoa butter chocolate, the regular consumption (14 days) of flavanol-rich milk chocolate (168 mg flavanols) was significantly linked with the reduction of SBP (−6 mmHg) and DBP (−5 mmHg), as well as with LDL cholesterol and oxidative stress markers in 28 healthy individuals (41). Similarly, short-term administration (7 days) of flavanol-rich dark chocolate significantly reduced SBP (*p* < 0.05) and insulin resistance (*p* < 0.001) in 20 healthy subjects (42). In 101 normotensive subjects, randomized to receive dark chocolate bars (397 mg flavanols), cocoa beverage (357 mg flavanols), or matching placebo for 6 weeks, the flavanol-rich chocolate consumption reduced SBP by 3.58 mmHg, with no significant effect on DBP (43). Furthermore, in 90 “healthy” elderly subjects, a statistically significant improvement in BP (*p* < 0.0001), insulin resistance (*p* < 0.0001), and lipid peroxidation (*p* = 0.001) were seen after 8 weeks in the high flavanol (993 mg, SBP: −7.83 ± 0.56 mmHg, DBP: −4.77 ± 0.37 mmHg) and intermediate flavanol (520 mg, SBP: −6.8 ± 0.59 mmHg, DBP: −3.2 ± 0.36 mmHg) intake groups in comparison to the low-flavanol group (48 mg, SBP: −1.6 ± 1.06 mmHg, DBP: −1.57 ± 0.61 mmHg). Moreover, flavanol consumption demonstrated a positive effect on cognitive performance (44). In the recently published Flaviola Health Study, 100 healthy subjects were enrolled and randomized to cocoa flavanol (CF) containing drink (450 mg) or CF-free drink for 1 month (27). CF intake decreased SBP and DBP by 4.4 mmHg (95% CI, 7.9–0.9 mmHg) and 3.9 mmHg (95% CI, 6.7–0.9 mmHg), improved endothelial function, and showed a positive effect on total and LDL cholesterol. By applying the Framingham Risk Score, CF intake significantly lowered the 10-year risk for CHD, CVD, and cardiovascular death in so-far healthy people (27). Moreover, Grassi and colleagues recently demonstrated that 1 week of supplementation with either 80 or 200 mg total flavanols (17 or 42 mg epicatechin, respectively) significantly decreased BP in healthy individuals (23). In particular, they documented a decrease in BP (SBP: −4.8 ± 1.03 mmHg, *p* < 0.0001; DBP: −3.03 ± 1.07 mmHg, *p* = 0.0011), and an improvement in endothelial function (23). However, in four different studies, flavanol-rich cocoa (234, 259, 550, 805 mg flavanols per day, respectively) did not improve BP levels compared to placebo in normotensive subjects (45–48).

The effect of cocoa consumption on BP was also assessed in hypertensive patients. In a randomized crossover trial, 13 hypertensive patients were randomized to dark polyphenol-rich chocolate (500 mg polyphenols) or white chocolate. After 14 days, only polyphenol-rich chocolate decreased SBP by 5.1 mmHg and DBP by 1.8 mmHg; after consumption discontinuation, BP levels returned to pre-intervention values within 2 days (49). Moreover, in 46 mildly hypertensive patients, low chronic cocoa intake (30 mg flavanols per day for 18 weeks) reduced SBP by −2.9 ± 1.6 mmHg (*p* < 0.001) and DBP by −1.9 ± 1.0 mmHg (*p* < 0.001) compared to placebo (50). In a crossover study, 20 subjects with never treated essential hypertension were randomized to dark (500 mg flavanols) or white chocolate (flavanol free) for 15 days. Dark chocolate decreased ambulatory BP (24-h SBP: −11.9 ± 7.7 mmHg, *p* < 0.0001; 24-h DBP: −8.5 ± 5.0 mmHg, *p* < 0.0001) (*p* < 0.0001), serum LDL cholesterol (*p* < 0.05), and improved vascular function (51). However, in a different crossover study enrolling 20 hypertensive subjects, flavanol-rich cocoa intake (900 mg per day) did not improve BP after 2 weeks, compared to placebo (52). A subsequent study evaluated the minimum dose required for BP lowering. A population of 52 subjects with untreated mild arterial hypertension was randomized to receive cocoa beverage containing different doses of flavanols (33, 372, 712, or 1,052 mg per day, respectively). After 6 weeks, only the highest flavanol dose (1,052 mg per day) demonstrated a significant reduction in 24-h SBP (5.3 ± 5.1 mmHg; *p* = 0.001), DBP (3 ± 3.2 mmHg; *p* = 0.002), and mean arterial BP (3.8 ± 3.2 mmHg; *p* = 0.0004) (53).

Flavanol intake also demonstrated a positive effect in hypertensive patients with impaired glucose tolerance (IGT) and diabetes mellitus. In particular, 19 hypertensive patients with IGT were randomized to receive flavanol-rich dark chocolate or flavanol-free white chocolate for 15 days. Dark chocolate reduced both SBP and DBP (SBP: −3.82 ± 2.40 mmHg; 24-h SBP: −4.52 ± 3.94 mmHg; DBP: −3.92 ± 1.98 mmHg; 24-h DBP: −4.17 ± 3.29 mmHg), total and LDL cholesterol (*p* < 0.0001) as well as improved vascular function and insulin sensitivity (*p* < 0.05) (54). High-flavanol cocoa (902 mg) reduced SBP and DBP (SBP: −1.9 mmHg, DBP: −1.8 mmHg, *p* < 0.05) and improved vascular function among overweight and obese adults (55). A recent study conducted on 60 subjects affected by hypertension and type 2 diabetes, randomized to flavanol-rich dark chocolate or white chocolate for 8 weeks, confirmed that flavanol-rich chocolate is effective in decreasing BP, fasting blood sugar, and triglyceride levels in patients with cardiovascular risk factors (28). However, in a high-risk population (three or more cardiovascular risk factors), flavanol-rich cocoa intake did not reduce BP values after 4 weeks (56).

Furthermore, in 24 heart failure patients, a 4 weeks consumption of high-flavanol dark chocolate (1,064 mg flavanols per day) significantly reduced DBP and NT-proBNP levels, compared to low-flavanol dark chocolate intake (57).

Several meta-analyses evaluated the available evidence on this topic. Ried and colleagues analyzed 13 studies, revealing a BP reduction after cocoa consumption (mean BP change ± SE: SBP: −3.2 ± 1.9 mmHg, *p* = 0.001; DBP: −2.0 ± 1.3 mmHg, *p* = 0.003). The effect maintained statistical significance only for the studies evaluating hypertensive and pre-hypertensive patients (SBP: −5.0 ± 3.0 mmHg; *p* = 0.0009; DBP: −2.7 ± 2.2 mmHg, *p* = 0.01) (58). Desch and coworkers also provided a meta-analysis including 10 randomized controlled trials involving either healthy subjects or patients with prehypertension/stage 1 hypertension (297 individuals). Cocoa consumption was associated with a 4.5 mmHg reduction (95% CI, −5.9 to −3.2, *p* < 0.001) for SBP and a 2.5 mmHg reduction (95% CI, −3.9 to −1.2, *p* < 0.001) for DBP (59). In the same year, a Cochrane review showed that flavanol-rich chocolate and cocoa products may have a statistically significant effect in lowering BP by 2–3 mmHg in the short term [SBP (95% CI): −2.77 mmHg (−4.72 to −0.82), *p* = 0.005; DBP (95% CI): −2.20 mmHg (−3.46 to −0.93), *p* = 0.006] (22). A recent published Cochrane review confirmed these findings, observing a small (2 mmHg) decrease in BP in the short term, more pronounced in the pre-hypertensive/hypertensive population (60).

## Cocoa and Vascular Function: Interventional Studies

Endothelium, the smooth, continuous inner lining of blood vessels, exhibits not only a barrier function but also synthetizes and releases a variety of vasoactive substances. Thus, the imbalance between vasodilating and vasoconstricting mediators results in endothelial dysfunction (61). The impairment of endothelial function is an early event in the development of atherosclerosis and is associated with CVD (62). Endothelial dysfunction of the forearm, as assessed by the flow-mediated dilation of the brachial artery (FMD), is recognized to be a powerful surrogate marker for cardiovascular events and cardiovascular mortality, both in healthy subjects (63) and in patients with CV risk factors (64). Thus, FMD has been used to evaluate the effects of different interventions on endothelial function. To date, various studies have subsequently assessed the effects of cocoa on vascular function (Table 2).

**Table 2:** Studies investigating cocoa and vascular function.

Reference	Year	Study design	Population	Duration	Intervention	Outcome
Rodriguez-Mateos et al. (65)	2015	Randomized double-blind crossover	15 healthy subjects	1,2,3,4 h	CF-rich drink (1.4–10.9 mg/kg body weight) vs. nitrate or nutrient-matched flavanol-free drink	Improvement in FMD after flavanol and nitrate intake

Engler et al. (46)	2004	Randomized double-blind placebo-controlled	21 heathy subjects	2 weeks	High flavonoid chocolate (213 mg procyanidins, 46 mg epicatechin) vs. low-flavonoid chocolate	Improvement in FMD, increased epicatechin concentrations

Sansone et al. (27)	2015	Randomized double-blind controlled parallel	100 healthy subjects	30 days	CF-containing drink (450 mg) or a nutrient-matched flavanol-free control bi-daily	Improvement in FMD, decreased SBP and DBP, decreased PWV

Grassi et al. (23)	2015	Randomized double-blind crossover controlled	20 healthy subjects	5 weeks	Five treatments with daily intake of 10 g cocoa (0, 80, 200, 500, 800 mg flavonoids)	Dose-dependent improvement in FMD, decreased PWV, and BP

Schroeter et al. (66)	2006	Randomized crossover	16 healthy subjects, isolated rabbit rings	2 h	Drink with high flavonoid content	Improvement in FMD, paralleled the appearance of flavanols in plasma

Heiss et al. (67)	2015	Randomized double-blind controlled parallel	42 healthy subjects	14 days	CF-containing drink (450 mg bid) vs. CF-free drink	Improvement in FMD, decreased PWV, and in total peripheral resistances

Shiina et al. (47)	2009	Randomized single-blind	39 healthy subjects	2 weeks	45 g commercially available dark chocolate vs. white chocolate	Improvement in coronary circulation as measured by coronary velocity flow reserve

Grassi et al. (51)	2005	Randomized crossover placebo-controlled	20 untreated hypertensive patients	15 days	100 g dark chocolate (21.91 mg catechin, 65,97 mg epicatechin) vs. flavanol-free white chocolate	Improvement in FMD, decreased BP and LDL cholesterol, increased insulin sensitivity

Grassi et al. (54)	2008	Randomized crossover placebo-controlled	19 hypertensive with IGT	15 days	100 g dark chocolate (36 mg catechin, 110 mg epicatechin) vs. flavanol-free white chocolate	Improvement in FMD, decreased SBP and DBP, decreased insulin resistance

Heiss et al. (69)	2005	Randomized double-blind crossover	11 smokers	2 h	100 ml cocoa drink with high (176–185 mg) or low (<11 mg) flavanol content	Improvement in FMD and increased circulating NO pool. Increased flavanol metabolites

Hermann et al. (70)	2006	Randomized placebo-controlled	20 smokers	2 h	40 g commercially available dark chocolate vs. white chocolate	Improvement in FMD, antioxidant status, and platelet function

Davison et al. (55)	2008	Randomized double-blind placebo-controlled parallel	49 obese and overweight patients	12 weeks	Dietary high (902 mg) vs. low (36 mg) flavanol intake	Improvement in FMD

Njike et al. (48)	2011	Randomized controlled crossover	44 overweight patients	6 weeks	Sugar-free cocoa beverage or placebo, sugar-sweetened cocoa beverage or placebo	Improvement in FMD, no change in weight

West et al. (71)	2014	Randomized double-blind crossover placebo-controlled	30 overweight patients	30 days	37 g dark chocolate plus sugar-free cocoa beverage (flavanols 814 mg) vs. low-flavanol chocolate bar and cocoa-free and sugar-free beverage	Unchanged FMD, increased basal diameter and peak diameter of the brachial artery, increased basal blood flow, in women decreased augmentation index

Balzer et al. (72)	2008	Randomized double-blind	41 diabetic patients	30 days	Flavanol-rich cocoa (321 mg flavanols × 3) or a nutrient-matched control (25 mg flavanols × 3)	Improvement in FMD

Mellor et al. (73)	2013	Randomized double-blind crossover controlled	10 diabetic patients	2 h	13.5 g of high vs. low-flavanol chocolate; 60 min later, a 75 g oral glucose load	Improved endothelial function assessed by reactive hyperemia peripheral artery tonometry

Heiss et al. (74)	2003	Randomized double-blind crossover	20 patients with at least 1 CV risk factor	2 h	Flavanol-rich cocoa drink (100 ml)	Improvement in FMD and increased levels of nitrosated and nitrosylated species

Heiss et al. (75)	2010	Randomized double-blind crossover controlled	16 CHD patients	30 days	Dietary high (375 mg bid) vs. low (9 mg bid) flavanol cocoa drink	Improvement in FMD and mobilization of endothelial progenitor cells

Flammer et al. (29)	2012	Randomized double-blind placebo-controlled	20 heart failure patients	2 h and 30 days	40 g commercially available dark chocolate vs. flavonoid-free placebo chocolate	Improvement in FMD of platelet function

Flammer et al. (76)	2007	Randomized double-blind	22 heart transplant patients	2 h	40 g commercially available dark chocolate vs. flavonoid-free placebo chocolate	Inducing coronary vasodilation, improvement in coronary endothelial function, and improvement in platelet function

Rassaf et al. (77)	2016	Randomized double-blind placebo-controlled	57 hemodialytic patients	30 days	CF-rich beverages (900 mg per study day) vs. flavanol-free beverages	Improvement in FMD decreased DBP. Ingestion of flavanols during HD alleviated HD-induced vascular dysfunction

Sansone et al. (68)	2017	Randomized double-blind crossover	47 healthy subjects		High (820 mg)/low-flavanol cocoa drink with high (220 mg)/low methylxanthines content	CFs with methylxanthines increased epicatechin serum concentration, increased FMD decreased PWV and DBP compared with flavanols alone

*Modified from Ref. (116)*.

*FMD, flow-mediated dilation; endoPAT, reactive hyperemia peripheral artery tonometry; PWV, pulse wave velocity; CV, cardiovascular; NO, nitric oxide; CHD, coronary heart disease; HD, hemodialysis; BP, blood pressure; SBP, systolic blood pressure; DBP, diastolic blood pressure; LDL, low-density lipoprotein; CF, cocoa flavanol; IGT, impaired glucose tolerance*.

In 15 healthy subjects, CFs intake (1.4–10.9 mg/kg body weight) acutely improved FMD at 1, 2, 3, and 4 h after consumption. The improvement in vascular function was comparable to the one induced by nitrate intake (65). Flavanol-rich chocolate (213 mg procyanidins, 46 mg epicatechin) consumption significantly improved FMD in 21 healthy subjects also over a 2-week period (*p* = 0.024). Moreover, plasma epicatechin concentrations markedly increased after 2 weeks in the active treatment group, suggesting that the effect on vascular function was flavanol-mediated (46). In the Flaviola Health Study, a 1-month CF intake increased FMD over control by 1.2% (95% CI, 1.0–1.4) and decreased pulse wave velocity by 0.4 m/s (95% CI, 0.8–0.04 m/s) (27). Furthermore, 20 healthy subjects were randomized to receive 5 treatments with daily intake of 10 g cocoa (0, 80, 200, 500, and 800 mg cocoa flavonoids per day) in5 periods lasting 1 week. A dose-dependent increase in FMD (from 6.2% to 7.3, 7.6, 8.1, 8.2% after the different flavonoid doses, respectively) was found (23). In healthy individuals, an improvement in vascular function after high-flavanol cocoa intake has been demonstrated in subsequent studies (66, 67). Flavanol-rich chocolate intake was also demonstrated to significantly improve coronary circulation in healthy adults, as assessed by coronary flow velocity reserve measurement with non-invasive transthoracic Doppler echocardiography (47). Interestingly, in a recently published randomized double-blind trial, an interaction between flavanols and methylxanthines, such as theobromine and caffeine contained in cocoa, has been identified. In particular, methylxanthines were demonstrated to increase epicatechin metabolites plasma levels, thus affecting flavanols absorption, and to enhance the positive vascular effect of flavanols (68).

Flavanol intake improved FMD in hypertensive patients with normal (51) as well as IGT (54). Flavanol-rich cocoa beverage also acutely improved endothelium-dependent vasodilation, platelet function, and circulating bioactive NO in smokers (69, 70). In 20 male smokers, dark chocolate, but not white chocolate, improved FMD after 2 h (7.0 vs. 4.4%, *p* = 0.026), and the effect lasted about 8 h after ingestion (70). Furthermore, a statistically significant reduction in DBP and mean BP, and a parallel improvement in FMD was shown after high-flavanol cocoa intake in obese and overweight subjects (55). Moreover, the positive effect of cocoa consumption on endothelial function was not associated with weight gain (48). Conversely, in a subsequent study, an increase in basal and peak brachial artery diameter, with no consequent change in FMD, was assessed in a group of 30 overweight patients after 30 days high-flavanol chocolate intake (71). In a diabetic population, high-flavanol cocoa consumption was associated with statistically significant improvement in vascular function both acutely (after 2 h) and chronically (30 days) in two different studies (72, 73). Flavanol-rich cocoa intake improved FMD (from 3.4 to 6.3%, *p* < 0.001) after 2 days in a population with at least one cardiovascular risk factor, including history of CHD, hypertension, hyperlipidemia, diabetes, or current tobacco use (74). Altogether, these studies highlight the ability of CFs in improving vascular function in patients with cardiovascular risk factors.

The efficacy of flavanol intake on vascular function has been assessed also in a population of 16 CHD patients, randomized to receive flavanol-rich or low-flavanol cocoa for 30 days. Results showed a significant improvement in endothelial function by 48%, a decrease in SBP (mean change in active group: −4.2 ± 2.7 mmHg), and increasing levels of circulating angiogenic cells in the active treatment group compared with controls (75).

In a study from our group, 20 chronic heart failure patients were randomized to receive commercially available flavanol-rich chocolate or control chocolate (29). Flavanol-rich chocolate significantly improved FMD from baseline levels of 4.98 ± 1.95, to 5.98 ± 2.32% (*p* = 0.045 and 0.02 for between-group changes) 2 h after intake, and to 6.86 ± 1.76% after 4 weeks of daily consumption (*p* = 0.03 and 0.004 for between groups). After flavanol-rich chocolate intake, platelet adhesion significantly decreased in the short term, but the effect was not sustained after 4 weeks. BP values and heart rate did not change. We also assessed the effect of flavanol-rich dark chocolate compared with control chocolate on coronary vascular function in 22 heart transplant recipients, patients characterized by severely impaired vascular function (76). Two hours after ingestion, flavanol-rich dark chocolate but not control chocolate induced significant coronary vasodilation (*p* < 0.01), improved coronary vascular function (*p* = 0.01), and decreased platelet adhesion.

Cocoa flavanols demonstrated to have a protective role also among patients with end-stage renal disease (ESRD). A population of 57 ESRD patients was randomized to receive either flavanol-rich beverage (900 mg) or placebo. Flavanol-rich cocoa improved endothelial function by 53% (*p* < 0.001) and alleviated hemodialysis induced endothelial dysfunction (*p* < 0.001) after acute ingestion, with no effect on BP and heart rate. After 4 weeks of treatment, cocoa improved vascular function by 18% and decreased DBP (*p* = 0.03) (77).

In a meta-analysis of 11 chronic and 11 acute studies, Hooper and colleagues found strong beneficial effects of cocoa on FMD, as well as reductions in DBP and mean arterial pressure. Chocolate or cocoa improved FMD regardless of the dose consumed (78).

Based on the epidemiological evidence and the results from interventional studies, the European Food Safety Authority (EFSA) published a health claim about the effect of polyphenols derived from cocoa on cardiovascular risk factors, assessing that CFs “help maintaining the elasticity of blood vessels, which contributes to normal blood flow.” In order to obtain the claimed effect, they suggested “to consume 200 mg of cocoa flavanols per day, provided by 2.5 g of high-flavanol cocoa powder or 10 g of high-flavanol dark chocolate, in the context of a balanced diet” (79). Conversely, Vlachojannis and coworkers asserted that only cocoa products with 100 mg epicatechin or CF doses of around 900 mg were able to achieve positive effects on FMD and BP, questioning the 200 mg flavanols/46 mg epicatechin dose recommended by the EFSA (80).

Currently, the COcoa Supplement and Multivitamin Outcomes Study (COSMOS; NCT02422745) is ongoing to assess the capability of a cocoa extract supplement (600 mg per day flavanols/80 mg epicatechin) compared to a standard multivitamin supplement, to reduce the risk of CVD and cancer among men aged 60 years and older and women aged 65 years and older. Concomitantly, an ancillary study (COSMOS-Mind; NCT03035201) is being conducted to evaluate the effects of such supplements on cognitive function.

## Putative Mechanisms

The protective role of CFs intake on BP and endothelial function is likely to come from its vasodilatory effect; the underlying mechanisms are multiple and not fully understood (30).

In this context, the increased NO availability and the subsequent vasodilation may play a central role. In young spontaneously hypertensive rats, epicatechin delayed the occurrence of arterial hypertension and reduced locomotor hyperactivity; these results were both mediated by increased NO bioavailability and erythrocyte deformability (81). Taubert and colleagues (50) demonstrated that prolonged intake of small amounts of dark chocolate (6.3 g per day for 18 weeks) reduced BP and improved NO production in a population of 44 pre-hypertensive individuals. In particular, dark chocolate intake reduced mean SBP by 2.9 mmHg (*p* < 0.001) and DBP by 1.9 mmHg (*p* < 0.001); these results were accompanied by a sustained increase of S-nitrosoglutathione, a source of bioavailable NO, by 0.23 nmol/L (*p* < 0.001) (50). Flavanols, and particularly flavanol-rich cocoa, elevate NO bioavailability by both stimulating the NO synthase (eNOS) activity (82, 83) and increasing the availability of l-arginine (*via* reduction of its degradation by arginase) (84). Furthermore, in a rat model, flavanols prevented the elevation of BP induced by l-nitroarginine methyl ester (l-NAME), a powerful inhibitor of NOS (83). In addition, cocoa showed a similar inhibitor effect on endothelin-1 production, a powerful vasoconstrictor (85, 86). Flavanols proved capable to induce both NO-mediated and endothelium-derived hyperpolarizing factor-mediated relaxation in a large number of arteries including the coronary arteries (87). Since NO degradation is mediated by free radicals, the improvement in vascular function is also related to the anti-inflammatory and antioxidant properties of cocoa (88). In a systematic review of the literature on polyphenols and oxidative stress, four studies reported statistically significant improvements in markers of oxidative stress and inflammation after flavanol-rich cocoa intake (89). A significant reduction in oxidative stress occurred when dark chocolate was administered to smokers as opposed to milk chocolate (90). Furthermore, a high dose (472.5 mg) of flavonoids through cocoa powder led to reduction in all oxidative and inflammatory markers in type 2 diabetics, and to a parallel improvement in endothelial function (1.7 ± 0.1 vs. 2.3 ± 0.1%, *p* = 0.01) assessed by reactive hyperemia peripheral artery tonometry (EndoPAT-2000) (73). After consumption of cocoa products, a decrease in markers of peroxidation was also observed in healthy subjects, as well as in obese, dyslipidemic, pre-hypertensive, and hypertensive patients (25). CFs directly scavenge ROS and nitrogen species (91); moreover, they modulate crucial enzymes related to oxidative stress, such as catalase, superoxide dismutase, glutathione peroxidase, glutathione reductase, glutathione transferase, xanthine oxidase, and lipooxygenase (92, 93). In line with this, the exposure of human endothelial cells to epicatechin inhibited endothelial NADPH oxidase, reduced superoxide and peroxynitrite levels, and consequently induced an increase in NO and cyclic guanosine monophosphate cellular levels (94). Furthermore, cocoa powder and epicatechin demonstrated to significantly decrease aortic oxidative stress and circulating markers related to impaired coagulation (von Willebrand factor, factor VIII, and fibrinogen) and inflammation (tissue necrosis factor-α, interleukin-6, interleukin-10, and C-reactive protein) (95). *In vitro* experiments show that CFs inhibit pro-inflammatory cytokines such as interleukin-2, interleukin-1β, and tissue necrosis factor-α, and positively modulate the expression of anti-inflammatory cytokines, such as interleukin-4 and transforming growth factor-β (92, 96–98).

An inhibition of angiotensin-converting enzyme (ACE) by cocoa constituents has been postulated. In a rat model, flavanol-rich cocoa powder significantly reduced BP in spontaneously hypertensive rats but did not exert a similar effect in normotensive Wistar-Kyoto rats. Interestingly, the effect of flavanol-rich cocoa (300 mg/kg) clearly mimicked that caused by captopril (50 mg/kg) (99). In 2006, Actis-Goretta and colleagues documented an *in vitro* interaction between cocoa flavonoids and ACE. They demonstrated that procyanidin-rich chocolate significantly inhibited purified ACE activity, whereas the inhibitory activity correlated with both the phenolic content (*p* < 0.003) and the flavanol content (*p* < 0.001). When incubated in membrane suspensions from rat kidney, chocolate [634 µM (+)-catechin equivalents] high in procyanidin inhibited ACE activity by 70% and such low in procyanidin [314 µM (+)-catechin equivalents] only inhibited ACE by 45% (*p* < 0.001). The inhibition of ACE in tissue membrane suspensions was also observed in rat testes and lungs (100). In a subsequent study, extract from powdered cocoa beans was demonstrated to dose-dependently inhibit *in vitro* ACE activity; it also showed a dose-dependent radicals scavenging ability (101). Persson and colleagues (102) demonstrated that the acute consumption of dark chocolate (75 g, 72% of cocoa) inhibited ACE activity *in vitro*—after incubation in human endothelial cells from umbilical veins (HUVEC) —and *in vivo* in 16 healthy volunteers. In HUVEC, a significant inhibition of ACE activity (*p* < 0.01) and an increase of NO levels (*p* < 0.001) were seen. In healthy subjects, dark chocolate significantly inhibited ACE activity (mean 18%) 3 h after oral intake, with no relevant changes in circulating NO levels. According to ACE genotype, significant inhibition of ACE activity emerged in individuals with genotype insertion/insertion and deletion/deletion (mean 21 and 28%, respectively) (102).

Recently, studies have begun to pay attention to the role of cocoa on mitochondria in cardiovascular health, as impaired mitochondrial function represents an early sign of endothelial dysfunction (103). The stimulation of mitochondrial function and biogenesis could ameliorate bioenergetic and metabolic status of cells, thereby improving vascular function and reducing CVD. Animal studies demonstrated that CFs are able to decrease cardiac post-ischemic damage *via* prevention of the mitochondrial permeability transition pore opening, and reduction in superoxide production (104); flavanols might also affect mitochondrial structure and function *via* stimulation of mitochondrial biogenesis (105). Patients, randomized to receive dark chocolate, showed increased maximal oxygen uptake and maximum work achieved, as well as increases in mitochondrial activity and glutathione levels, when compared to placebo (106). NO is suspected to mediate the effects of cocoa on mitochondria. In support of this thesis, a recent study demonstrated, *in vitro*, that flavanols are capable to stimulate mitochondrial function and biogenesis; effects disappeared with the inhibition of eNOS (107, 108).

Cocoa flavanols are also able to inhibit platelet activation, adhesion, and aggregation, mechanisms that play a central role in the development of endothelial dysfunction and atherosclerosis (109). Indeed, activated platelets secrete a number of adhesion molecules, such as P-selectin and C40 ligand, release inflammatory mediators into the local microenvironment (110), stimulate the chemotaxis of leukocytes to the site of inflammation (111), and generate ROS, reducing NO bioavailability and contributing to endothelial dysfunction and thrombosis (112).

Moreover, cocoa and its main flavanols may improve vascular function by regulating the glucose and lipid profile (113), crucial risk factors for vascular damage. There is evidence that CFs are able to modulate insulin secretion in β-pancreatic cells, target insulin-sensitive tissues, repress glucose production, enhance glucose uptake through the promotion of glucose transport, and improve lipid metabolism (114).

**Figure 1:**
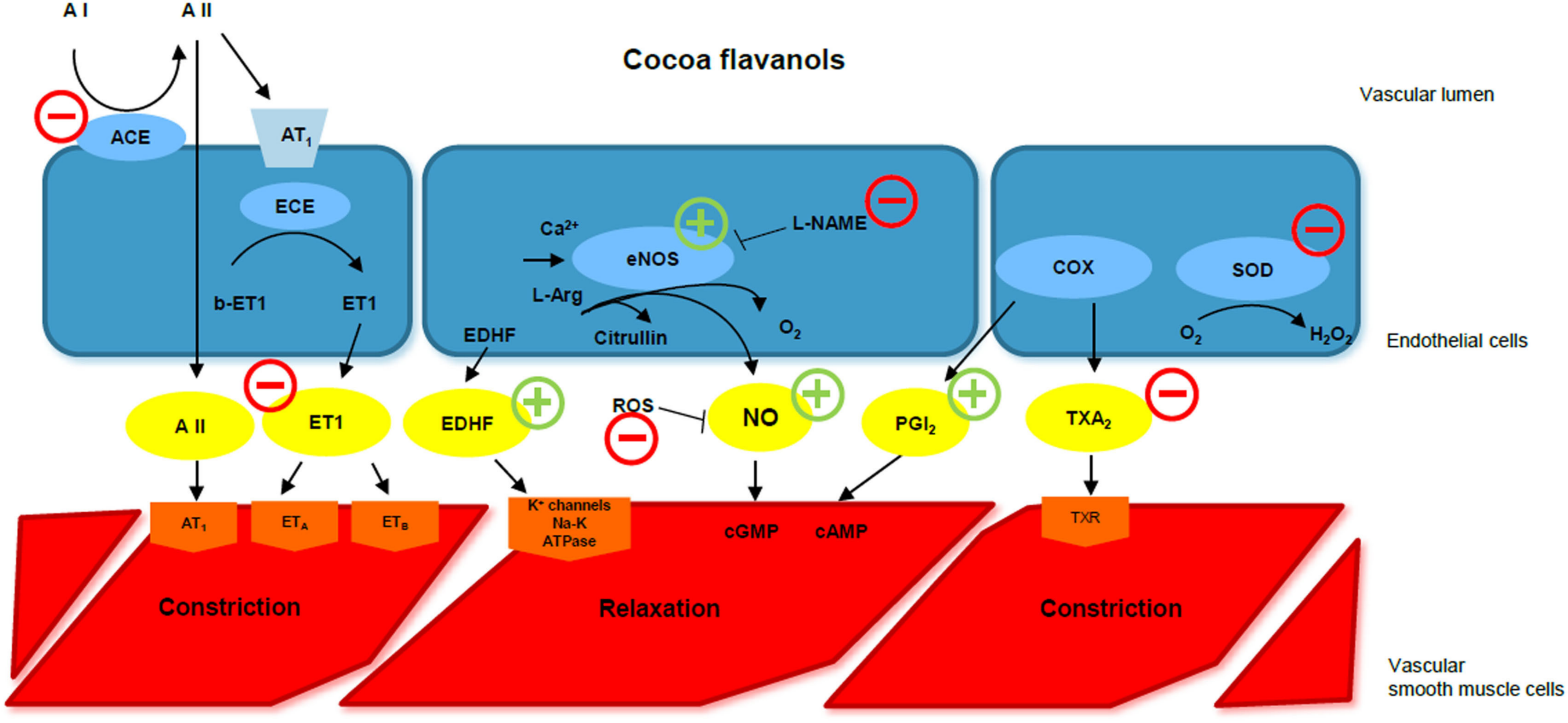
Cocoa flavanols and endothelial function. ACE, angiotensin-converting enzyme; A I, angiotensin I; A II, angiotensin II; ECE, endothelin-converting enzyme; ET 1, endothelin-1; EDHF, endothelium-derived hyperpolarizing factor; eNOS, endothelial NO synthase; l-NAME, l-nitroarginine methyl ester; ROS, reactive oxygen species; NO, nitric oxide; cGMP, cyclic guanosine monophosphate; cAMP, cyclic adenosine monophosphate; COX, cyclooxygenase; PGI2, prostacyclin; TXA2, thromboxane A2; SOD, superoxide dismutase; CF, cocoa flavanol. CFs improve endothelial function *via* different pathways. They increase NO availability, stimulating eNOS function, preventing l-NAME-induced hypertension, and reducing ROS. They also stimulate EDHF-mediated relaxation, inhibit endothelin-1, and reduce ACE activity. Modified by Corti et al., Circulation 2009.

Altogether, these mechanisms might determine the antihypertensive and cardiovascular protective effects of flavanols *in vivo* (Figure 1).

## Conclusion

Polyphenol-rich foods, such as fruit, vegetables, wine, olive oil, and cocoa, are able to reduce cardiovascular risk and prevent cardiovascular events and death (3–5, 115). Among them, cocoa beans have always been of particular interest, as they are one of the richest polyphenol sources. In this context, several epidemiological studies suggest a strong correlation between daily cocoa intake and better cardiovascular outcome in different population settings (19–21). Clinical interventional studies demonstrated a positive effect of flavanol-rich cocoa or chocolate intake on BP reduction and improvement in microvascular and macrovascular function (116, 117). *In vitro* and *in vivo* studies identified increased NO availability, increased NO synthase activity, and inhibition of ACE as putative mechanisms of this beneficial effect (118).

Cocoa consumption has been demonstrated to improve endothelial function and to lower BP in healthy subjects, in patients with risk factors and hypertension, and in patients with coronary heart disease and heart failure. Furthermore, a 3-mmHg reduction in SBP has been estimated to lower the relative risk of CHD by 5% and the risk of global mortality by 4% (119). Thus, the introduction of a moderate amount of flavanol-rich cocoa in the daily diet may be a promising strategy to improve cardiovascular outcomes.

However, commercial chocolate with its high sugar and fat content may be undesirable in a population with increased cardiovascular risk. Furthermore, during the cocoa beans manufacturing process, the total amount of flavanols can be reduced more than 10-fold by fermentation or roasting (18). This results in an unpredictable content of polyphenols in most commercially available products. Moreover, the optimal dose of daily flavanol intake is still unclear. The EFSA recommends consuming 200 mg of CFs per day, provided by 2.5 g of high-flavanol cocoa powder or 10 g of high-flavanol dark chocolate in the context of a balanced diet, but this dose has recently been subject of discussion (80). Thus, a comparison among studies is difficult, because of the heterogeneity between trials, in terms of study population and design, flavanol doses in active and control groups, and study duration.

Further clinical studies are required in order to identify the correct dose and the right modality of manufacturing of flavanol-rich cocoa to be able to benefit from daily consumption of this natural medicinal product in the field of CVD.

## Author Contributions

All authors made substantial contributions to the conception of this review. VL, JB, MN, and IS drafted the work; FE, CF, AF, and FR revised it critically for important intellectual content. All authors approved the final version of the paper.

## Conflict of Interest Statement

The authors declare that the research was conducted in the absence of any commercial or financial relationships that could be construed as a potential conflict of interest.
